# Automated Cell Foreground–Background Segmentation with Phase-Contrast Microscopy Images: An Alternative to Machine Learning Segmentation Methods with Small-Scale Data

**DOI:** 10.3390/bioengineering9020081

**Published:** 2022-02-18

**Authors:** Guochang Ye, Mehmet Kaya

**Affiliations:** Department of Biomedical and Chemical Engineering and Sciences, Florida Institute of Technology, 150 W University Blvd, Melbourne, FL 32901, USA; gye2014@my.fit.edu

**Keywords:** automation, cell culture, computer vision, image analysis, optical microscopy, deep learning

## Abstract

Cell segmentation is a critical step for image-based experimental analysis. Existing cell segmentation methods are neither entirely automated nor perform well under basic laboratory microscopy. This study proposes an efficient and automated cell segmentation method involving morphological operations to automatically achieve cell segmentation for phase-contrast microscopes. Manual/visual counting of cell segmentation serves as the control group (156 images as ground truth) to evaluate the proposed method’s performance. The proposed technology’s adaptive performance is assessed at varying conditions, including artificial blurriness, illumination, and image size. Compared to the Trainable Weka Segmentation method, the Empirical Gradient Threshold method, and the ilastik segmentation software, the proposed method achieved better segmentation accuracy (dice coefficient: 90.07, IoU: 82.16%, and 6.51% as the average relative error on measuring cell area). The proposed method also has good reliability, even under unfavored imaging conditions at which manual labeling or human intervention is inefficient. Additionally, similar degrees of segmentation accuracy were confirmed when the ground truth data and the generated data from the proposed method were applied individually to train modified U-Net models (16848 images). These results demonstrated good accuracy and high practicality of the proposed cell segmentation method with phase-contrast microscopy image data.

## 1. Introduction

In a cell culture laboratory, checking the cells under the microscope is a daily routine. Based on the image observed under a microscope, experienced researchers can only have an approximated sense about the confluence of culturing cells, the morphology of the cells, and if contamination happens [1]. Appropriate steps are taken to maintain the cell line growth depending on all this visual information. As a critical parameter in adherent monolayer cell culture, cell proliferation rate is directly reflected via changes in the cell growth area. Plenty of biotechnological protocols, such as Alamar blue [2], MTT (3-[4,5-dimethylthiazol-2-yl]-2,5-diphenyltetrazolium bromide) [3], and XTT (sodium 3´-[1-(phenylaminocarbonyl)-3,4-tetrazolium]-bis(4-methoxy6-nitro)benzene sulfonic acid hydrate) [4], are used to measure this parameter indirectly, but these methods lean on costly instruments and professionally trained technicians. They are highly invasive or require cell staining, which disturbs the growth of the cell or even causes the termination of cell culture [5]. If these experimental conditions are not always available, the visual inspection of cell images under the microscope can be a solution, followed by manual segmentation of cell clusters. This tedious task is challenging when the number of samples is tremendous or the input complexity is counterintuitive. Additionally, it is unavoidable that these manual measurements are prone to subjective errors when a lab technician does not have sufficient training. Alternatively, advanced computer vision approaches could be implemented to achieve analysis functions accurately and efficiently [6]. 

Cell segmentation is a critical process in computer vision applications for microscopy image analysis and recognition [6,7]. Besides the standard edge detection methods, including Sobel, Canny, Prewitt, Roberts, and fuzzy logic methods [8,9,10], researchers currently use machine learning techniques for cell segmentation and characterization on histopathology samples [11,12,13,14]. Microscope images are often affected by uneven illumination caused by a lens or lamp. This issue causes a bright central area and some shading areas towards the corners [15]. During the monolayer cell culture, dead cells or unhealthy cells can lose the adhesive state and leave the lens focus plane. The location change in z-direction appears like a giant halo on the microscope image. These noisy microscope images compromise the existent techniques’ performance. As a popular software to handle image processing in biological studies, ImageJ (the National Institutes of Health, Bethesda, USA and the Laboratory for Optical and Computational Instrumentation (LOCI), University of Wisconsin, Madison, WI, USA) provides convenient macros and plugins for cell segmentation. However, the task requires users to repeat the same measurement on every single cell image, which is performed by circling the target cell by hand with an embedded drawing tool [16]. These techniques may also require additional human effort to fine-tune them. Hence, a cell segmentation method at a high automatic level is still needed.

Several efforts in cell segmentation have been made to automate all the image processing tasks [10,13,17,18,19]. Nevertheless, human input or hand-tuning is still essential to achieve decent accuracy. Featured with high flexibility and accuracy predictions, the performances of machine learning techniques are believed to surpass conventional image processing techniques. However, current deep learning techniques [20,21] for cell segmentation unavoidably require a sufficiently large amount of labeled image data in the training phase, for which it is time-consuming to label a sufficiently large amount of image data. Furthermore, the introduced subjective errors during manual labeling may hinder the performance of machine learning techniques. To provide accurate cell segmentation and to automate data preparation for machine learning techniques, this study proposes an accurate and practical image processing method to achieve cell segmentation for phase-contrast images with minimization of human efforts. It applies grayscale morphology operators to extract the cellular edge information instead of using existing popular edge detection methods, such as Canny [22]. The Otsu threshold technique [23] is applied here, since this unique binary threshold technique does not require users to provide a specific threshold value. The optimal binary threshold value is generated by minimizing the between-class variance [24]. With the resulting binary image, a couple of binary morphological operators are used sequentially for removing the noise. The input image is separated into two images; the foreground image contains only the growth cell. The other image is the background image, which does not include cells. The cell growth area is calculated by dividing the total number of pixels of the foreground cell image by the total number of pixels of the input image. Dice coefficient and Intersection over Union (IoU) are calculated for assessing the similarity between the binary masks generated from the proposed method and the ground truth.

## 2. Theory

**Figure 1:**
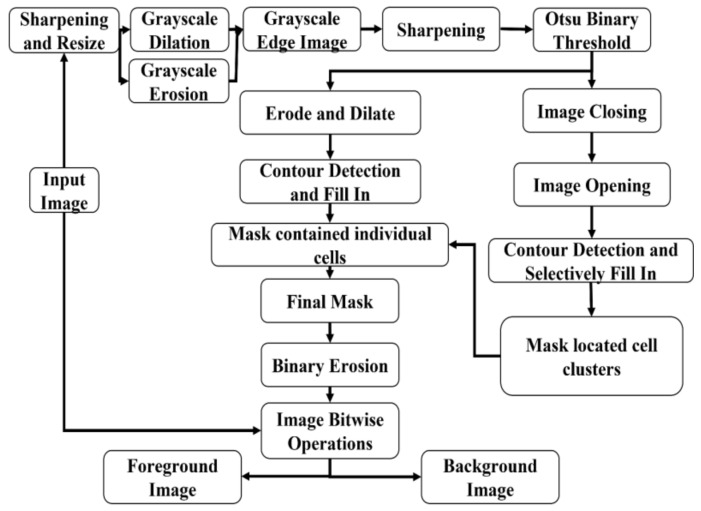
The flowchart of the proposed method.

The original input image was resized to 600 × 800 pixels and converted to the grayscale format. A Gaussian blur (kernel size is 3) was applied for removing high-frequency noise. The resulting image underwent morphological erosion and dilation on the grayscale level with a 3 × 3 kernel. The external-edge image was obtained by subtracting the resulting image before any morphological operation from the dilated image. Additionally, the internal-edge image was obtained by subtracting the eroded image from the resulting image before any morphological operation. Finally, the mid-edge image was obtained by subtracting the eroded image from the dilated image. All three images containing edge information were added and combined as an edge image. After applying Gaussian blur and image sharpening techniques, the Otsu binary threshold was applied to this edge image [23]. Contours were detected and filled in as solid blobs on the edge image to generate one image mask that contains small-size individual cells or partially attached cells. The previous edge image was processed again through morphological closing, followed by opening with a rectangular structuring element in a size of 9 × 9 pixels for locating cell clusters. All the detected cell clusters or fully attached singular cells were located. When the tiny gap regions are found within the detected contours, that specific gap region would be classified as a background without filling within the cellular contour when an average pixel value of a gap region was closer to the average pixel value of the detected background regions. Otherwise, that region would be filled and classified as a cellular foreground. The two mask images were added and combined as the final mask image. This resulting mask image was used for image masking to separate the input image into two parts, the foreground image (with cells on it) and the background image (with no cells found on it). A flowchart for the illustration of the proposed method is shown in Figure 1.

The Gaussian Blur can be described as:

(1) $$G\left(x,y\right)=\frac{1}{2{\pi \sigma }^{2}}e^{-\frac{x^{2}+y^{2}}{2\sigma^{2}}}$$

where σ is the standard deviation of the Gaussian distribution, x is the distance from the origin in the horizontal axis, and y is the distance from the origin in the vertical axis.

The grayscale morphological operations can be described as:

(2) $$\mathrm{Dilation}:\;{\left[f\;⊕D\right]}_{\left(x\right)}=\max\;\left\{f\left(Z\right)\;:Z\in D_{x}\right\}$$

(3) $$\mathrm{Erosion}:\;{\left[f⊖D\right]}_{\left(x\right)}=\min\left\{f\left(Z\right)\;:Z\in D_{x}\right\}$$

where ⊕ is the image dilation operation, ⊖ is the image erosion operation, f is the input image, and D is the structuring element (SE). For an arbitrary 2D grayscale input image (f), the central element of an SE (D) is mapping on the input image one by one pixel (from left to right, from up and down). During each mapping, all the pixels, which fall into the SE, form a pixel set. These pixel positions are included in *Z*. For the grayscale dilation, the maximum pixel value is found from this set and assigned to the current mapping pixel; for the grayscale dilation, the minimum pixel value is chosen instead. The binary morphological operations can be described as:

(4) $$\mathrm{Binary}\;\mathrm{Dilation}:{\;\mathrm{pixel}}_{\mathrm{ij}}=\left\{\begin{array}{c} 255,\;\;\;\;\;\;\mathrm{if}\;\mathrm{any}\;\mathrm{pixel}\;\mathrm{value}\;\mathrm{of}\;255\;\mathrm{exits}\;\mathrm{in}\;\mathrm{the}\;\mathrm{SE}\\ 0,\;\;\;\;\;\;\mathrm{if}\;\mathrm{none}\;\mathrm{of}\;\mathrm{pixel}\;\mathrm{value}\;\mathrm{with}\;255\;\mathrm{in}\;\mathrm{the}\;\mathrm{SE} \end{array}\right.$$

(5) $$\mathrm{Binary}\;\mathrm{Erosion}:{\;\mathrm{pixel}}_{\mathrm{ij}}=\left\{\begin{array}{c} 0,\;\;\;\;\;\;\mathrm{if}\;\mathrm{any}\;\mathrm{pixel}\;\mathrm{value}\;\mathrm{of}\;0\;\mathrm{exits}\;\mathrm{in}\;\mathrm{the}\;\mathrm{SE}\\ 255,\;\;if\;none\;of\;pixel\;value\;with\;0\;in\;the\;SE \end{array}\right.$$

Similar to the grayscale dilation, for an arbitrary binary input image, if any pixel that falls in the SE is binary high (255), the pixel value corresponding to the central element is assigned with 255; otherwise, it is 0 (this means all the pixel values are zeros inside the SE). For binary erosion, the pixel value corresponding to the central element is assigned with 0 if any pixel that falls in the SE is binary low (0).

(6) $${\mathrm{Edge}}_{\mathrm{external}}={\left[f\;⊕D\right]}_{\left(x\right)}-f_{\left(x\right)}$$

(7) $${\mathrm{Edge}}_{\mathrm{middle}}={\left[f\;⊕D\right]}_{\left(x\right)}-{\left[f⊖D\right]}_{\left(x\right)}$$

(8) $${\mathrm{Edge}}_{\mathrm{internal}}={\;f}_{\left(x\right)}-{\left[f⊖D\right]}_{\left(x\right)}$$

(9) $${\mathrm{Edge}}_{\mathrm{all}}={\;\mathrm{Edge}}_{\mathrm{external}}+{\mathrm{Edge}}_{\mathrm{middle}}+{\mathrm{Edge}}_{\mathrm{internal}}$$

The external edge is obtained from image subtraction between the dilated image and the original image for the edge information extraction. The subtraction between the dilated and eroded images yields to the middle edge. The internal edge is obtained by subtracting the eroded image from the original image. All the edges are summed up for the following analysis in the proposed method. 

Once the edge information is extracted, the image can be binarized into two main components (edge pixels and non-edge pixels) with the Otsu threshold method. The optimal binary threshold value is calculated from:

(10) $$\mathrm{Binary}\;\mathrm{Threshold}\;\mathrm{Value}=\mathrm{argmin}\;\left(W_{\mathrm{background}}\sigma_{\mathrm{background}}^{2}+W_{\mathrm{foreground}}\sigma_{\mathrm{foreground}}^{2}\right)$$

where σ^2^ is the variance and W is the weight for each component. The weight W of one component is calculated by the number of pixels in this component divided by the input image’s total number of pixels. 

Among this work, the relative error is used biasedly to reveal the accuracy of cell growth measurement. Dice coefficient and Intersection over Union are calculated for assessing the similarity between the binary masks generated from the proposed method and the ground truth binary masks. These parameters are calculated by the following equations:

Relative Error = Abs (Measured–Actual)/Actual(11)

(12) $$\mathrm{Dice}\;\mathrm{Coef}=2*\left(X\cap Y\right)/\left(\left|X\right|+\left|Y\right|\right)$$

(13) $$\mathrm{Intersection}\;\mathrm{over}\;\mathrm{Union}\;=\left(X\cap Y\right)/\left(X\cup Y\right)$$

X and Y in (12) and (13) are binary images.

## 3. Materials and Methods

All the data (156 images) were phase-contrast microscope images obtained from Woodworth’s lab (Clarkson University, Potsdam, NY, USA). The image sizes of the data were identical, with 1392 × 1040 pixels. Among these image data, multiple cells were represented. These include cells from the human ectocervix zone, endocervix zone cultured in vitro, and the human papillomavirus (HPVs, involving HPV16 E6 and E7) immortalized human cervical transformation zone cells. The cell differentiation was induced in the medium with 1.4 mM calcium chloride to induce differentiation [25]. Under the induction, multiple cell morphologies were shown to vary in size and shape, and cell debris was easily spotted. All the pictures had different distributions of cell growth among the different cell types. All the cellular contours were drawn manually with the assistance of the LiveWire function on the ImageJ software [26], which served as the control group (ground truth) to validate the performance of the proposed method. Trainable Weka Segmentation (TWS), as a popular unsupervised machine learning tool, achieves segmentation via pixel classification [27]. The ilastik 1.3.3 (S. Berg, D. Kutra, T. Kroeger, C. N. Straehle, B. X. Kausler, C. Haubold, M. Schiegg, J. Ales, T. Beier, M. Rudy, K. Eren, J. I. Cervantes, B. Xu, F. Beuttenmueller, A. Wolny, C. Zhang, U. Koethe, F. A. Hamprecht, and A. Kreshuk, Heidelberg, Germany), as an open-source software for image classification, performs segmentation and pixel classification with a random forest classifier [28]. The Empirical Gradient Threshold (EGT) method, as a novel image gradient threshold selection method, featured automated segmentation across image modalities and cell lines with imaging processing techniques [29]. These three recent advanced methods were used for comparison and validation purposes.

### 3.1. Proof of Concept

The overall segmentation accuracy was validated as follows: for each input image, one binary mask resulting from the proposed method was obtained for calculating dice coefficient, IoU, and cell growth area against its ground truth binary image. Due to the limited space, two of the phase-contrast microscopy images of human cervical cells were used in this section as samples for simple demonstrations. One image was taken at low cell density and the other image was taken at high cell density. The cell morphology was more diversified on the low-density image than the high-density image. The resulting images from the selected pipeline steps are shown in the results section.

### 3.2. Effect of Input Image Blurriness

Images taken from an improperly calibrating microscope were conferred with a certain degree of blurriness, which also happened with the low-quality lens or under misfocusing lens adjustments. The blurriness caused the image data to be inapplicable to manual labeling methods. For confirming the reliability of the proposed method and quantitating the effect of image blurriness on the accuracy of cell segmentation during the proposed procedures, image blurriness methods (the Gaussian blurriness and the mean blurriness) were applied to the input images with varying kernel sizes, ranging from 3 to 49. Dice coefficient, IoU, and relative error on cell growth area were measured in every kernel size at both blurring methods. 

### 3.3. Effect of Input Image Size

Images with small sizes are unable to carry much detailed information. However, a large-size image is cumbersome to handle and the noise components (such as cell debris) are certainly magnified. The proposed method consisted of basic imaging processing steps. Thus, the size of the input image (the number of pixels) directly affects method runtime and the accuracy of image operations (including opening, closing, dilation, and erosion). To verify the reliability and the independent accuracy performance of the proposed method to the varying input image’s size, a set of scalars (ranging from 0.5 to 3, the scalar was applied to image height and image width at the same time) were generated for expanding and shrinking the image size. The size of an input image was increased maximally by nine times and scaled down to one-fourth of the preset input size (800 × 600 pixels) with the same aspect ratio. Dice coefficient, IoU, and relative error on cell growth area measurements at each condition are presented in the results section.

### 3.4. Effect of Image Illumination

The light condition is directly linked to image pixel intensities, and dark images are complicated or unusable in manual segmentation methods. This section explored the adaptive performance and the independent accuracy performance of the proposed method under varying illumination. Two methods were used for adjusting image illumination. The first method was directly multiplying a value (after subtracting a scalar from 255) with the input image, which was already normalized between 0 and 1. The other method involved directly subtracting a scalar from each pixel of the input image. In both of these illumination adjusting methods, scalars ranged from 0 to 150, with a step of 5. Hence, the maximum pixel of the adjusted input images would ideally be in the range of 105 to 255. For each condition, dice coefficient, IoU, and relative error on cell growth area measurements are presented in the results section.

### 3.5. Validation with U-Net Deep Learning Model

**Figure 2:**
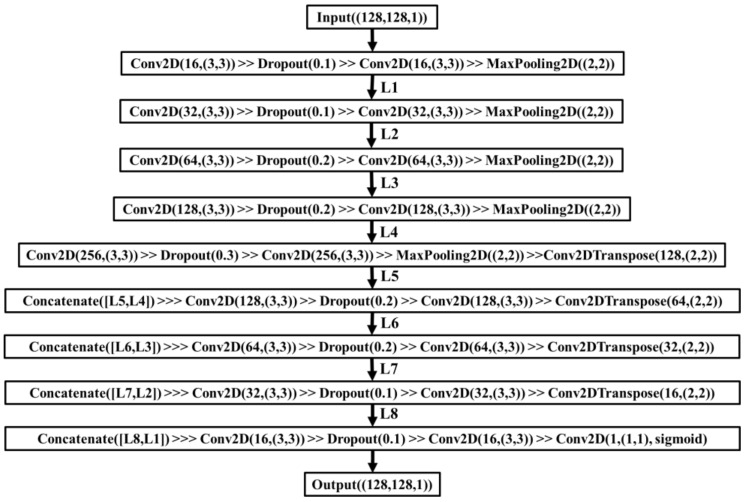
The model architecture of the modified U-Net used in this study.

In this section, a modified U-Net deep learning model inspired by [30,31] was trained with manually labeled binary data (ground truth) and the resulting binary images from the proposed cell segmentation to further validate the proposed method. The model architecture is shown in Figure 2. All the cellular regions were marked with white color with black backgrounds. To thoroughly review the performance of the learning model, each image (total of 156 images) went through the sliding window algorithm (window size is 128 × 128 pixels, with a 64-pixel sliding step on x and y directions) to expand the size of the dataset (total of 16848 images in each group). The size of the input to this model was 128 × 128 pixels. In the training phase, the optimizer was set to Adam (learning rate = 5 × 10^−5^), and a soft dice loss function (epsilon = 1 × 10^−6^) was set as a compiling loss function. Dice coefficient and IoU were selected as metrics. Training epoch was set to 50 epochs, with early stopping (by monitoring the loss parameters of each epoch) enabled to prevent model overfitting. For each dataset, 70% of images were used for training the model, and each dataset generated one model. For comparing these two model performances, the remaining 30% of images were used as the inputs. Dice coefficient, IoU, and relative error on cell growth area measurements were calculated between the predicted binary mask and the ground truth to demonstrate the accuracy achieved by the proposed cell segmentation method.

### 3.6. Computational Tools

The image processing code was written using Python 3.6 (Python Software Foundation, Wilmington, DE, USA). The OpenCV (Intel Corporation, Santa Clara, CA, USA) [32] was used as the main library for image processing, including finding contours of the image and handling morphological operations on binary images. Meanwhile, the SciPy library [33] was used to handle morphological operations on grayscale images. The NumPy library was utilized for managing the image sharpening [34]. Keras [35] with TensorFlow [36] backend was mainly supporting the machine learning section. An input image was the only human input at this proposed segmentation method. 

## 4. Results

**Figure 3:**
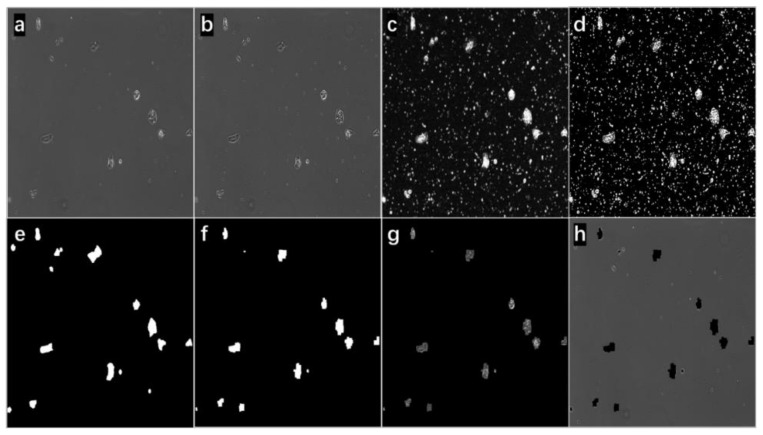
The cell image with low-density input. (**a**) The input image (from Input Image in Figure 1); (**b**) the sharpened image (from Sharpening and Resize in Figure 1); (**c**) the image contained all the edge information (from Grayscale Edge Image in Figure 1); (**d**) the edge image (from Otsu Binary Threshold in Figure 1); (**e**) the manually labeled binary image for comparison here; (**f**) the cell region binary mask; (**g**) the foreground image; (**h**) the background image.

**Figure 4:**
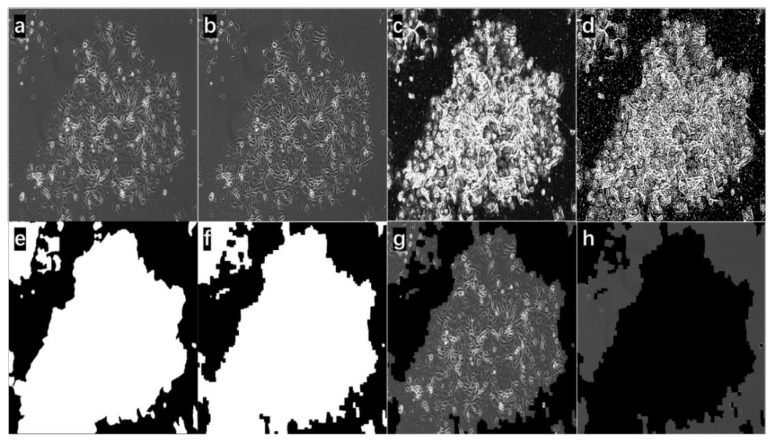
Cell image with high-density input. (**a**) The input image (from Input Image in Figure 1); (**b**) the sharpened image (from Sharpening and Resize in Figure 1); (**c**) the image contained all the edge information (from Grayscale Edge Image in Figure 1); (**d**) the edge image (from Otsu Binary Threshold in Figure 1); (**e**) the manually labeled binary image for comparison here; (**f**) the cell region binary mask; (**g**) the foreground image; (**h**) the background image.

As a simple demonstration, eight images resulting from the selected steps of the proposed method at two confluence conditions (high cell density and low cell density) were used. In Figure 3, part a is the original input image, and the cells are at low density compared with Figure 4a. Some of them were not fully attached to the tissue culture flask and appeared as tiny bright circles. The compromised image quality and the ‘fuzziness’ of the image background were some of the challenges of the project. Figure 3c is the edge image obtained from grayscale morphological operations. The edge was dim and thin. After applying a sharpening kernel, the edge was intensified. The Otsu binary threshold technique was applied to the image in Figure 3c to obtain Figure 3d; then, the main contours were detected and conditionally filled in as solid blobs. Figure 3f shows all the cellular regions, and Figure 3e displays the manually labeled cellular contours serving as the ground truth for the purpose of comparison. With the bitwise operations, the foreground image (Figure 3g) and the background image (Figure 3h) were obtained. By comparing part a with part g, the proposed method can achieve cell segmentation with good accuracy in this case. Nearly all the cellular structures were detected. On Figure 3h, only an unnoticeable number of cellular structures was found; cellular debris and halo artifacts (at least 10 places) that appeared on the image are certainly excluded as uncountable information. Similar accuracy segmentation results were observed again on a high-density cell image (in Figure 4). The same approach was taken to obtain the resulting images. By comparing part a with part h in Figure 4, nearly no cellular structures were left. On Figure 4g, some narrow area that was noncellular was detected successfully, which is difficult to be identified during segmentation manually. Additionally, these results supported that the advantage of applying this proposed automated segmentation method was to largely prevent subjective errors from manual segmentation. 

After producing binary segmentation masks for all 156 images via the proposed method, (Table 1) these resulting masks achieve 90.07% (std: 3.93%) as the average dice coefficient, 82.16% (std: 6.33%) as the average IoU, and 6.51% (std: 5.04%) as the average relative error on measuring cell growth area. This achieved accuracy outperformed the TWS method (81.54% as dice coefficient, 69.46% as IoU, and 36.00% as relative error), the EGT method (85.79% as dice coefficient, 76.07% as IoU, and 31.47% as relative error), and the ilastik software (87.32% as dice coefficient, 77.84% as IoU, and 22.80% as relative error). In the TWS method, small-sized cell debris floating around as the background was mostly detected undesirably, and the processes required more user intervention and time. The EGT method’s efficiency is comparable to the proposed method, but it requires users to fine-tune the setting, especially when the image artifacts exist. The ilastik software achieved better accuracy than the TWS and the EGT methods, though this method required the most computation time. With the proposed method, it took 8.72 seconds to process all the images (average of 56 milliseconds per image), which is superbly efficient compared to manual segmentation (about 10–20 min per ground truth image, depending on the complexity of the cell region distribution). Therefore, the results generated by our method were much closer to the ground truth, and the cell segmentation results were achieved with high overall accuracy and high efficiency at a highly automatic level.

**Figure 5:**
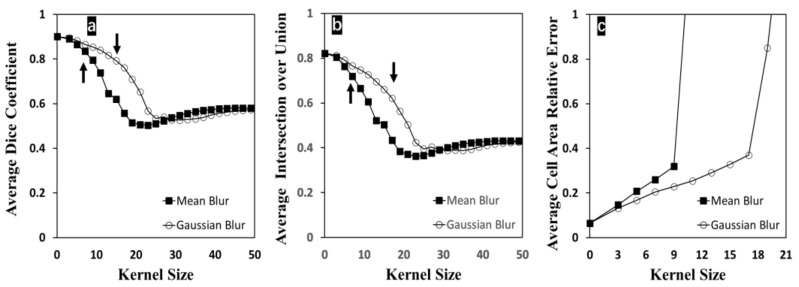
Effect of input image blurriness on the proposed cell segmentation method. (**a**) Dice coefficient changed with respect to increasing kernel size; (**b**) IoU changed with respect to increasing kernel size; (**c**) relative error on cell growth area measurements changed with respect to increasing kernel size. Dark arrows indicate the conditions that manual segmentation is infeasible.

In Figure 5, average dice coefficient, IoU, and relative error on cell growth area measurements were plotted with changing kernel size during blurring. The degree of blurriness is increased with increasing the size of the kernel. Once the kernel size reached 7 in the mean method or 17 in the Gaussian method, the majority of edge information was lost and the input image would not be applicable for manual segmentation. In Figure 5a and Figure 5b, dice coefficient and IoU began to decrease with increasing kernel and reached their minimum at around 23 as the kernel size in the mean method or at about 31 as the kernel size in the Gaussian method. After these points, both parameters maintained steady (approximately 0.55 for dice coefficient and 0.43 for IoU). When the kernel size exceeded these minimum points, the proposed segmentation method could not behave functionally; these high degrees of blurriness resulted in the cell region detection failures by defectively affecting the edge detecting step. In Figure 5c, when the kernel size was larger than 19, the relative error was more than 100% with respect to the manually labeled ground truth data. The relative errors at kernel size larger than 21 were not shown here because the cell area measurement with an unacceptably high error becomes meaningless. Compared to dice coefficient and IoU, the cell growth area measurement was only considering the size of the detected area without including the location of the detected pixels. When the images suffered from a severe blurring issue, the cellular contours, outlined with cleared edges under the normal condition, now would be detected approximately with softened edges, or multiple separated objects would be detected as one connected object, which contributed to unwantedly increasing the cell growth area. Thus, this metric was shown to be more sensitive to the degree of blurriness. By comparing these two blurring methods, the proposed method was more sensitive to the mean blurred images and showed more tolerance to the Gaussian blurriness at the same kernel size. However, this proposed method, consisting of conventional image processing techniques, is prone to be affected by the image quality. With a kernel size of 9 in the mean blur method, the cell regions of the input image are unidentifiable for humans. In contrast, the proposed method can still achieve cell segmentation with decent accuracy (the average dice coefficient is 79.53% and the average IoU is 66.56%), which confirms the outstanding reliability of the proposed method compared to the manual labeling.

**Figure 6:**
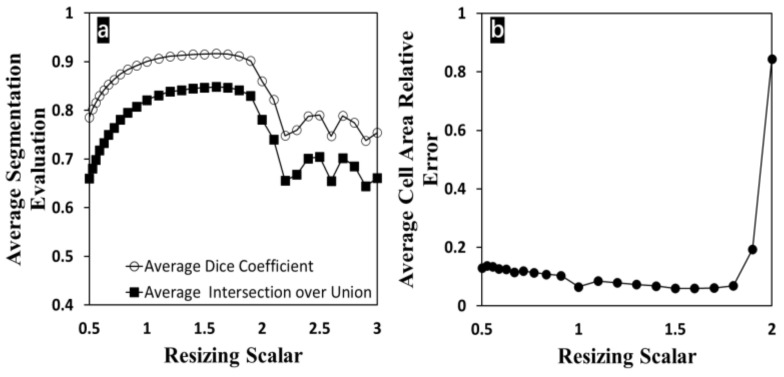
All the cell image sizes were modified with scalars ranging from 0.5 to 3 (marked on the x-axis). The image aspect ratio is unchanged. (**a**) Dice coefficient and IoU changed with increasing scalars; (**b**) relative error on cell growth area measurements changed with increasing scalars.

Besides the blurriness effect, as seen in Figure 6, similar reliable performance of the proposed method has been observed again with changing input image sizes. Within the scalar range, the image size ranged from 300 × 400 pixels (one fourth of the original size with a scalar of 0.5) to 1800 × 2400 pixels (nine times the original size with a scalar of 3). The original image size was found unchanged at the scalar of 1. In Figure 6a, the dice coefficient and IoU were found barely unchanged between 0.77 (87.49% as dice coefficient; 78.16% as IoU) and 1.90 (90.18% as dice coefficient; 83.02% as IoU) as resizing scalars. A similar steady trend was observed when resizing scalars were between 0.77 and 1.8 on measuring the cell growth area. In Figure 6b, the cell area relative error showed more tolerance to the changing image size and exceeded 100% error after expanding the image with 2 as the scalar. The relative errors at scalars larger than 2 were not shown here because the cell area measurement with an unacceptably high error becomes meaningless. With increasing the size of the image, noise components (cell debris, image artifact) become considerable and included in the segmentation, which reduces the dice coefficient and IoU. To mitigate this issue, a large image (18 times larger than the original size) can be resized to 800 × 600 pixels and input to the proposed method, resulting in 87.54% as the dice coefficient and 78.09% as IoU. In the case of image shrinking, details (edge information) of the image tend to be lost; some of the cell regions now are too small to be detected completely and could be removed from the morphological operations, which also reduce the dice coefficient and IoU. However, these two unwanted conditions will unfavorably hinder the segmentation accuracy. Within the range from 60% to 360% of the original image size, the high accuracy performance of the proposed method is independent of the input size. The proposed segmentation method is shown to have outstanding reliability covering an extensive range of image resolutions.

**Figure 7:**
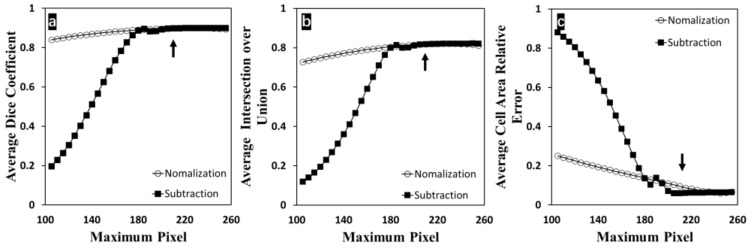
Effect of input image illumination on the proposed cell segmentation method. (**a**) Dice coefficient changed with varying illumination; (**b**) IoU changed with varying illumination; (**c**) relative error on cell growth area measurements changed with respect to varying illumination. Dark arrows indicate the conditions that manual segmentation is infeasible.

As seen in Figure 7, reliable and accurate performance was observed when varying illumination conditions were introduced to the input images. In Figure 7a and Figure 7b, dice coefficient and IoU were maintained steadily for both light-adjusting methods before the maximum pixel value fell from 255 to 180. Within this range, dice coefficient ranged from 89.02% to 90.07% and IoU ranged from 80.43% to 82.15% in the normalization group; dice coefficient ranged from 88.36% to 90.09% and IoU ranged from 80.03% to 82.19% in the directed subtraction group. When the maximum pixel of the input image was reduced below 180 during adjusting illumination, the dice coefficient and IoU decreased heavily in the directed subtraction group, but decreased only mildly in the normalization group. The directed subtraction method is similar to thresholding, and image information is lost regardless. In contrast, the normalization method is similar to rescaling the image in the bit-depth conversion. This method could better maintain the image contrast and edge information relatively than the directed subtraction method. Thus, the proposed method showed more overall tolerance to the normalization method for image illumination adjusting. In Figure 7c, cellular area relative error increased more slowly within the range from 190 to 255 in the directed subtraction group than the normalization group. When the maximum pixel kept decreasing from 190, the error of the directed subtraction group increased faster than the normalization group. Normally, cellular structures are in a dark tone, while the background pixels are in a bright tone. Within the range from 190 to 255, in the direction subtraction group, some pixels from the cell regions were set entirely to zeros to remove some noise caused by these low-value pixels. However, the direction subtraction method is not good at maintaining the image contrast. The image sharpening step among the proposed segmentation method can effectively compensate for this contrast loss so that the cell area relative errors could be kept lower than the normalization group. In addition, it is worth mentioning that manual segmentation methods become infeasible when the maximal pixel value is reduced to 210 after illumination adjusting in the directed subtraction group. The pixel range was shrinking to 0 to 210, and almost one sixth of bit-depth information was missing. The darkening image upscaled the upfront difficulties and reduced the segmentation accuracy or the efficiency during manual labeling. Compared to these supporting results, the proposed method could perform cell segmentation with good reliability and accuracy, even when the maximal pixel value was reduced to 180, which is inapplicable to the manual methods.

**Figure 8:**
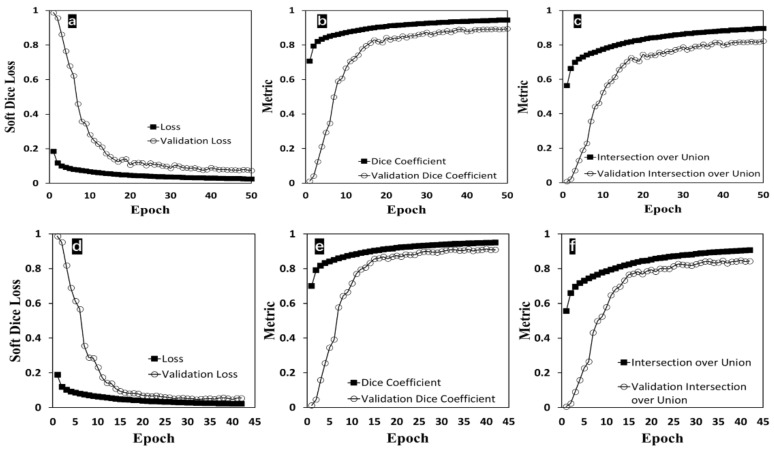
U-Net model training with 10% of training data as the validation. (**a**) Soft dice loss function with respect to epoch; (**b**) dice coefficient as the metric with respect to epoch; (**c**) IoU as the metric with respect to epoch (a, b, and c are from the model training with the binary results from this proposed cell segmentation method). (**d**) soft dice loss function with respect to epoch; (**e**) dice coefficient as the metric with respect to epoch; (**f**) IoU as the metric with respect to epoch (d, e, and f are from the model training with the ground truth data generated from manually labeling).

For additionally validating the accuracy of segmentation results from the proposed method, the resulting binary images set and the ground truth binary images set were used to train modified U-Net models. In Figure 8, soft dice loss function and two metrics (dice coefficient and IoU) had mostly identical trends between the experimental group and the ground truth group. After finishing model training, predictions were made from these two models by inputting the same set of test cellular images. All two sets of predictions were compared to the ground truth data generated via manual labeling. In Table 2, the ground truth model, trained with the ground truth data, achieved 95.01% as the dice coefficient and 90.99% as IoU compared to the target data (originally from the ground truth data). The experimental model, trained with the resulting binary images from the proposed method, achieved 92.16% as the dice coefficient and 86.42% as IoU when the predicted binary images were compared to the target data used in the ground truth model (originally from the ground truth data). The predictions, made from two U-Net models trained with two different datasets, had a high degree of similarity; this indirectly reflects the high accuracy of the proposed cell segmentation method compared to the ground truth data.

As expected, the proposed segmentation method yielded a similar accuracy compared to the trained U-Net model. The proposed method achieved 91.20% as the dice coefficient and 84.87% as IoU compared to the target data used in the ground truth model. The proposed method’s accuracy performance is comparable to the U-Net method in this study. Furthermore, these machine learning results firmly supported that this proposed cell segmentation method could provide an automatic way of generating accurate binary labeled image data for cell segmentation neural network training.

## 5. Discussion

In this study, we developed a simple but effective cell segmentation method consisting of fundamental image processing techniques. This method achieved high accuracy in segmenting the human cervical cell images compared to the most recent methods (WTS, EGT, and the ilastik software). Even under the extreme conditions under which manual segmentation becomes unfeasible and time-consuming, along with high sensitivity to subjective errors, this method maintained adaptive reliability facing blurriness, darkening, and resolution compromising. Additionally, predictions with high similarity resulting from the experimental and ground truth U-Net models also validated the accuracy of the proposed segmentation method.

Existing approaches (such as the WTS) provide easy installation, and users could also operate them as plugins on the ImageJ platform. However, extended amounts of time are required to explore the segmentation settings or to manually label the segmentation areas, which is additionally required to train the machine-learning/deep-learning-based segmentation methods (e.g., U-Net model). Differently, the proposed method provides more accurate cell segmentation with minimal user intervention, as shown in the results section. In the proposed approach, the edge information was efficiently extracted via a series of grayscale morphology operations instead of using conventional edge detection methods (e.g., Canny). The Otsu method was chosen intentionally as it achieves the binarization on the cellular edge information image instead of the whole cellular grayscale image. These two steps assured the high-level automation and efficiency of the proposed method. The following image processing step handled the cell cluster regions and the single individual cell regions separately, securing the segmentation accuracy. Combining efficient functions, operations, or steps resulted in our processing method requiring less computation demand, which is another advantage over the existing methods. Additionally, this model is intuitive to being adjusted for the users without expertise in image processing. When needed, the proposed pipeline could be easily modified to adapt to a new imaging setting; the proposed method’s flexibility and practicality are valuable features. As the limitation of this study, the proposed pipeline works explicitly with phase-contrast images. However, phase-contrast images are the most common type of microscopy data. Therefore, this highly automatic level segmentation method will be beneficial for countless bioengineering related researchers for their cellular image analysis or data preparation/preprocessing in the development of machine/deep learning models.

Recently, there was another related study to measure the cell confluence [37]; the cell segmentation method in that study relies on the image texture homogeneity quantified by the standard deviation of pixel intensities. They assumed the cellular regions had a more inhomogeneous texture than the noncellular background. After the texture analysis, the directed threshold filter was applied, and the threshold value was determined uniquely through iterative visual inspections for each given cell type. Cellular foregrounds were detected after analyzing each small region on the input images. In [38], the cell segmentation data was automatically generated from fluorescent images and performed similarly to manual annotations in deep convolutional neural networks training. Our study is different to them, since segmentation was performed on native cell images without staining and the edge of the cellular regions was obtained by simply grayscale morphological operations instead of using other conventional edge detection methods (e.g., the Canny algorithm), which lean on users’ fine-tune and are hard to be automated among varying image conditions (e.g., illumination). The cellular edge information was mainly focused and processed for foreground–background separation instead of relying solely on image regions or pixel intensities. As the advantages of this work, the proposed method here requires less user intervention and is reliable even under unfavorable image conditions, and its accuracy segmenting output is completely unaffected by the determined threshold values during edge extraction.

In the cellular segmentation field, the functional principle or algorithms of existing software (e.g., CKX-CCSW Confluency Checker [39] and CellProfiler [40]) are difficult to assess by the users, which can hinder users from fine-tuning the image processing parameters based on their actual needs [27]. Inappropriate software setting always compromise the segmentation accuracy. As an open-source approach, instead of using conventional image processing, machine learning techniques have become popular in this field and successful in segmenting varying types of cellular images, including medical images [41,42,43]. DeLTA, a deep learning model, provides cell segmentation and tracking at the same time and has performed cellular analysis for Escherichia coli cells inside a microfluidic device [44]. U-Net, another deep learning model, achieved good accuracy segmentation (92.03% as IoU) on PhC-U373 dataset on the 2015 ISBI cell tracking challenge [31], and this deep learning method has been modified and broadly applied to medical imaging segmentation [45,46,47]. These machine learning approaches have been demonstrated with better adaptive performance compared to the conventional imaging processing techniques on cell segmentation when sufficient training data are available. In contrast, the development of conventional image processing for handling segmentation on small-scale data has seldom been reported in recent years. Thus, this study practically provides an alternative cell segmentation path with image processing when the machine learning methods are not feasible on small-scale data. Meanwhile, this work also presents an excellent solution to accurately automate data preparation in a fast and reliable manner for segmentation model development on label-free phase-contrast image data with the use of deep learning models. In some other deep learning methods (e.g., StarDist [48], CellPose [49]) that would detect cells on the individual cell level, the proposed segmentation method will also be beneficial for researchers to apply as a critical step for data preprocessing (foreground–background segmentation) for mostly eliminating unfavored noise coming from the noncellular background to potentially improve the accuracy and efficiency of these cell-by-cell segmentation methods [50]. Furthermore, the proposed method is lightweight, which can be executed smoothly on minicomputers, such as Raspberry Pi, directly connected to the low-cost microscope camera to measure the cell growth area at a real-time pace. This low-cost setup can primarily protect the cell confluence assessment from human errors.

## 6. Conclusions

In conclusion, this study introduces an efficient algorithm that performs better than existing methods (WTS, EGT, and the ilastik software) in terms of accuracy, computational time, level of automation, flexibility, and practicality for foreground/background segmentation of phase-contrast images. The reliability of the proposed segmentation method was investigated and observed at varying limited conditions, including adding artificial blurriness, adjusting illumination, and changing image size. In addition, it provided a convenient way for generating accurately labeled image data for promoting machine/deep learning techniques applied to cell segmentation and analysis. Therefore, the researchers related to bioengineering and biomedical sciences would benefit from the outcomes of this study for their cellular image analysis or data preparation/preprocessing, especially in the development of machine/deep learning models.

## Figures and Tables

**Table 1:** Segmentation performance with different methods.

Method	Dice Coefficient (%)	IoU (%)	Relative Error (%)
Proposed	90.07	82.16	6.51
TWS	81.54	69.46	36.00
EGT	85.79	76.07	31.47
Ilastik	87.32	77.84	22.80

**Table 2:** Model performance with U-Net models trained with different segmentation data.

Models	Dice Coefficient (%)	IoU (%)
Ground truth	95.01	90.99
Experimental	92.16	86.42

## Data Availability

Usage of all the image data in this work is sustained under the material transfer agreement between Florida Institute of Tech, FL, USA and Clarkson University, NY, USA.
